# Resolving synaptic events using subsynaptically targeted GCaMP8 variants

**DOI:** 10.1101/2025.06.19.660577

**Published:** 2025-06-19

**Authors:** Jiawen Chen, Junhao Lin, Kaikai He, Luyi Wang, Yifu Han, Chengjie Qiu, Dion Dickman

**Affiliations:** 1 University of Southern California, Department of Neurobiology, Los Angeles, CA USA; 2 USC Neuroscience Graduate Program

**Keywords:** synapse, calcium imaging, GCaMP, Drosophila, neuromuscular junction

## Abstract

While genetically encoded Ca^2+^ indicators are valuable for visualizing neural activity, their speed and sensitivity have had limited performance when compared to chemical dyes and electrophysiology, particularly at synaptic compartments. We addressed these limitations by engineering a suite of next-generation GCaMP8-based indicators, targeted to presynaptic boutons, active zones, and postsynaptic compartments at the Drosophila neuromuscular junction. We first validated these sensors to be superior to previous versions. Next, we developed a new Python-based analysis program, *CaFire,* which enables the automated quantification of evoked and spontaneous Ca^2+^ signals. Using *CaFire,* we show a ratiometric presynaptic GCaMP8m sensor accurately captures physiologically-relevant presynaptic Ca^2+^ changes with superior sensitivity and similar kinetics compared to chemical dyes. Moreover, we test the ability of an active zone-targeted, ratiometric GCaMP8f sensor to report differences in Ca2+ between release sites. Finally, a newly engineered postsynaptic GCaMP8m, positioned near glutamate receptors, detects quantal events with temporal and signal resolution comparable to electrophysiological recordings. These next generation indicators and analytical methods demonstrate that GCaMP8 sensors, targeted to synaptic compartments, can now achieve the speed and sensitivity necessary to resolve Ca^2+^ dynamics at levels previously only attainable with chemical dyes or electrophysiology.

## INTRODUCTION

Evolution’s ingenuity is evident in its exploitation of the calcium ion (Ca^2+^) as a potent secondary messenger, essential for controlling a diverse array of physiological events in every cell. The basis for Ca^2+^’s extraordinary signaling capacity lies in a formidable concentration gradient – maintained at nanomolar levels inside cells versus millimolar outside – that exceeds a thousand-fold. This carefully maintained difference allows for controlled Ca^2+^ entry to rapidly amplify cytosolic Ca^2+^ concentrations, in turn activating specific Ca^2+^-sensitive proteins to initiate vital downstream signaling pathways (Berridge et al., 2003, Clapham, 2007). In neurons, this Ca^2+^-driven control system achieves a pinnacle of sophistication. The powerful Ca^2+^ gradient is harnessed to orchestrate crucial neuronal processes, from shaping synaptic strength at dendritic spines and modulating gene expression in response to activity, to the precise triggering of neurotransmitter release via synaptic vesicle (Dittman et al., 2019, Jackman et al., 2017, McCarthy et al., 2024). Therefore, the ability to directly observe and interpret these dynamic Ca^2+^ signals in neurons provides a crucial lens, offering profound insights into the complex cellular operations that define neuronal function and flow of information through neural circuits.

Given the paramount importance of Ca^2+^ signaling in virtually all aspects of neuronal function, an extensive series of genetically-encoded Ca^2+^ indicators (GECIs) have been engineered with progressively enhanced speed and sensitivity. The archetypal GECI, GCaMP, ingeniously utilized a modified green fluorescence protein (GFP) flanked by the Ca^2+^ binding protein Calmodulin (CaM) and a Ca^2+^/CaM-binding M13-like peptide (Nakai et al., 2001). Upon Ca^2+^ binding, a conformational change in the CaM-peptide interaction allosterically modulates GFP fluorescence. While a conceptual breakthrough, early GCaMP iterations suffered from relatively slow kinetics, limited dynamic range, and modest fluorescence changes (Tian et al., 2009). Consequently, a variety of sophisticated protein engineering strategies were employed to bolster GCaMP performance, leading to significant improvements represented in the GCaMP6 and GCaMP7 series, which offered faster kinetics and higher signal-to-noise ratios (SNRs) (Dana et al., 2019, Chen et al., 2013). Finally, the subsequent development of the GCaMP8 series marked a substantial leap forward (Zhang et al., 2023). This enhanced performance was not merely incremental; it resulted from a deep understanding of the molecular mechanisms underlying GCaMP activation, involving meticulous optimization of the CaM/peptide interface for faster Ca^2+^ association and dissociation and other improvements that led to enhanced brightness and efficient conformational coupling (Zhang et al., 2024). These comprehensive engineering efforts yielded GCaMP8 variants that exhibit dramatically improved kinetics – both in rise and decay times – and sensitivity relative to previous generations. Indeed, their performance characteristics, including SNR, response speed, photostability, and linearity, became comparable or even superior to some of the best synthetic Ca^2+^ dyes, while retaining the crucial advantage of genetic targetability (Zhang et al., 2024). These innovations provide an opportunity to resolve Ca^2+^ dynamics at subcellular compartments with unprecedented resolution.

Many studies express GCaMP variants alone in the neuronal cytoplasm, a method that effectively captures the large, relatively slow Ca^2+^ fluxes in soma that correlate with action potential spiking (Akerboom et al., 2012, Peron et al., 2015, Ziv et al., 2013). However, Ca^2+^ transients within subcellular compartments, such as dendritic spines and presynaptic terminals, are characterized by significantly faster kinetics and occur within smaller, more restricted volumes than those in the general cytoplasm, posing distinct measurement challenges. Importantly, interpreting signals from GCaMPs expressed alone can be confounded by variations in sensor abundance due to uneven expression levels, differing protein stability, or photobleaching, any of which can obscure the accurate determination of true Ca^2+^ changes (McMahon et al., 2018). Ratiometric indicators are therefore an attractive alternative, as they typically involve co-expression of Ca^2+^-sensitive and Ca^2+^-insensitive fluorescent proteins in a stoichiometric manner (Zhang et al., 2021). By normalizing the Ca^2+^-dependent signal to the Ca^2+^-independent one, ratiometric measurements can control for these variations in sensor concentration and provide a more reliable quantification of Ca^2+^ levels and dynamics. Hence, an optimal approach for accurately capturing fast and local subsynaptic Ca^2+^ fluxes would target an advanced, ratiometric GCaMP sensor, engineered for high sensitivity and rapid kinetics, to the particular compartment being investigated.

The Drosophila neuromuscular junction (NMJ) is a powerful and genetically tractable model system for dissecting Ca^2+^ signaling dynamics at a glutamatergic synapse. Its utility is magnified by the ability to combine sophisticated genetic manipulation with GECIs, advanced imaging, and established electrophysiological approaches to monitor synaptic events with remarkable spatiotemporal resolution (Bellen et al., 2010, Frank et al., 2013). Early investigations into Ca^2+^ dynamics at the fly NMJ employed chemical dyes (e.g., OGB-1, Fura-2), which, despite their rapid kinetics, present challenges in controlling loading concentrations at synaptic compartments (Macleod et al., 2002). The advent of GECIs offered an opportunity for targeted and consistent expression at subcellular regions. Pioneering engineering of presynaptic GECIs enabled Ca^2+^ visualization at axonal boutons or active zones (Kiragasi et al., 2017, Cohn et al., 2015, Akbergenova et al., 2018), but were often non-ratiometric for Ca^2+^ sensing – complicating quantification – and employed older GCaMP variants with suboptimal kinetics and SNRs. Postsynaptic sensors evolved from using GCaMP2 to GCaMP6f, offering better sensitivity for detecting quantal events (Newman et al., 2017, Peled et al., 2014). More recently, GCaMP8f variants were employed (Perry et al., 2022, Li et al., 2021, Han et al., 2023), demonstrating promising sensitivity. However, other studies suggested that GCaMP8m may offer higher SNRs and similar kinetics, particularly in Drosophila (Zhang et al., 2023). These developments motivated us to engineer and validate optimal GECIs for the fly NMJ, aiming for performance that rivals chemical dyes for presynaptic imaging and electrophysiology for postsynaptic quantal event detection.

We have engineered and characterized a variety of next-generation GECI probes to resolve Ca^2+^ dynamics at pre- and post-synaptic compartments at the Drosophila NMJ. While an expected tradeoff between signal strength and kinetics was observed, GCaMP8m consistently delivered the highest responses with only modest reductions in speed relative to GCaMP8f. Notably, presynaptic GCaMP8m sensors captured physiologically important events with speed and sensitivity approaching that of chemical dyes, while postsynaptic GCaMP8m probes achieved quantal event detection with similar resolution to electrophysiological recordings. These features indicate targeted ratiometric GCaMP8m sensors and CaFire analysis as complementary tools for resolving synaptic events using purely optical approaches.

## RESULTS

### Engineering next generation GCaMP sensors targeted to synaptic compartments

**Figure 1:**
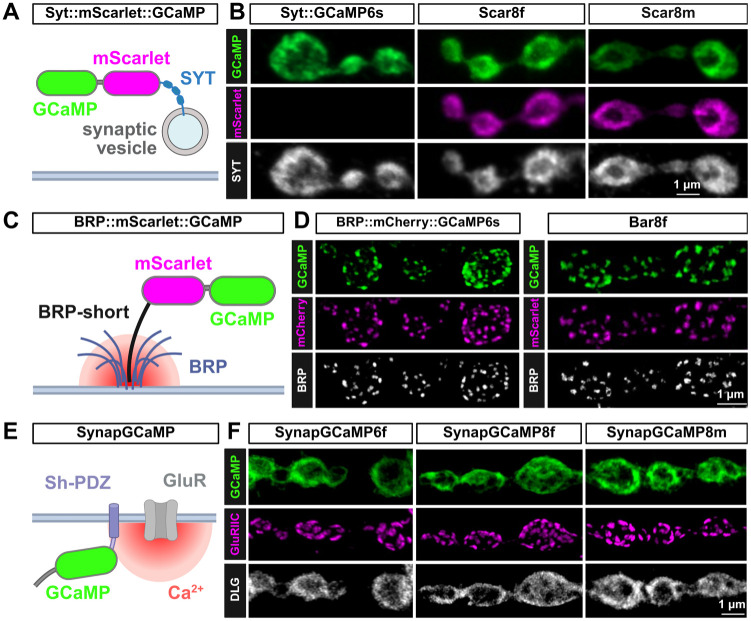
GCaMP indicators targeted to pre- and post-synaptic compartments. **(A)** Schematic of the presynaptic ratiometric Syt::mScarlet::GCaMP (Scar8f/Scar8m) Ca^2+^ indicators showing localization to synaptic vesicles via fusion to the Ca^2+^ sensor Synaptotagmin (SYT). **(B)** Representative images of NMJs expressing the indicated reporter driven in motor neurons with the OK319-GAL4 driver (*w*^*1118*^; *OK319-GAL4/UAS-Scar8f*) immunostained with anti-GFP (GCaMP) and anti-SYT. Note that endogenous mScarlet signals were obtained without antibody labeling. **(C)** Schematic of the BRP::mScarlet::GCaMP8f (Bar8f) ratiometric Ca^2+^ indicator, which targets GCaMP to active zones via fusion to the BRP-short protein (Schmid et al., 2008). **(D)** Representative images of NMJs expressing the indicated reporter driven in motor neurons (*w*;*OK319-GAL4/Bar8f*) immunostained with anti-GFP (GCaMP) and anti-BRP. Note that native mCherry or mScarlet signals were obtained without antibody labeling. **(E)** Schematic of the SynapGCaMP indicator, which targets GCaMP to postsynaptic compartments via a Shaker PDZ domain.(Newman et al., 2017) **(F)** Representative NMJs expressing the indicated reporter (*w*;*MHC-CD8-GCaMP6f-Sh/+*;*+*, *w*;;*MHC-CD8-GCaMP8f-Sh/+*, *w*;;*MHC-CD8-GCaMP8m-Sh/+*) immunostained with anti-GFP (GCaMP), - GluRIIC (glutamate receptors), and -DLG (postsynaptic density).

To engineer the next generation of synaptically-targeted GCaMP sensors, we sought to improve upon previous sensors employed for Ca^2+^ imaging at the Drosophila NMJ. In particular, we focused on three cassettes. First, to target GCaMP to presynaptic boutons, GCaMP6s was fused to the synaptic vesicle protein Synaptotagmin (SYT), generating UAS-SYT::GCaMP6s (Cohn et al., 2015), schematized in Fig. 1A. More recently, this SYT::GCaMP fusion strategy was improved upon by replacing GCaMP6s with the faster and more sensitive GCaMP8f, and rendered ratiometric by fusion to the red-shifted, monomeric fluorescent protein mScarlet (Bindels et al., 2017), to generate UAS-SYT::mScarlet::GCaMP8f (Scar8f) (Li et al., 2021) (Fig. 1A). To potentially improve this indicator, we replaced GCaMP8f with GCaMP8m, which is reported to exhibit higher sensitivity with only minor reductions in kinetics (Zhang et al., 2023), and replaced mScartlet1 with mScarlet3, which encode improvements in brightness and photophysical characteristics (Gadella et al., 2023), to make the ratiometric GECI Scar8m (Fig. 1A). Expression of each sensor with the motor neuron driver OK319-Gal4 and immunostaining demonstrated the expected trafficking to presynaptic boutons and co-localization with SYT (Fig. 1B).

Next, to target GCaMP to the sites of presynaptic Ca^2+^ influx, active zones (AZs), we improved upon a previous indicator, UAS-BRPshort::mCherry::GCaMP6s (Kiragasi et al., 2017). This strategy used a “short” fragment of the AZ scaffold Bruchpilot (BRP) (Schmid et al., 2008), which traffics to AZs, fused to mCherry and GCaMP6s for ratiometric Ca^2+^ imaging at AZs (Fig. 1C). To improve upon this design, we replaced mCherry with the improved mScarlet, and GCaMP6s with GCaMP8f to make UAS-BRPshort::mScarlet::GCaMP8f (Bar8f) (Fig. 1C). Expression of these sensors in motor neurons followed by immunostaining also confirmed the expected trafficking to AZs and co-localization with BRP (Fig. 1D). Finally, we worked to improve upon a postsynaptic Ca^2+^ sensor. A previous cassette called SynapGCaMP6f fused GCaMP6f to a Shaker PDZ motif to target the sensor near postsynaptic NMJ glutamate receptors (GluRs) under the control of a muscle-specific enhancer to enable “quantal imaging” of single synaptic vesicle release events (Newman et al., 2017) (Fig. 1E). A recent improvement replaced GCaMP6f with GCaMP8f (Han et al., 2022), and here we engineered GCaMP8m to make SynapGCaMP8m (Fig. 1E). Immunostaining of NMJs expressing each sensor confirmed proper trafficking and localization to postsynaptic densities near GluRs (Fig. 1F). In addition, GCaMP is a Ca^2+^ buffer and may therefore impact synaptic development and/or function (McMahon et al., 2018). Thus, we recorded from NMJs expressing the indicators introduced above, and found that expression of the GCaMP sensors did not perturb synaptic transmission, with the except of Scar8f, which showed a moderate reduction in EPSP amplitudes (Fig. S1). Collectively, these tools established the foundation of our study to go on to benchmark each sensor and determine the optimal indicator, and their speed and sensitivity, to resolve pre- and post-synaptic Ca^2+^ events and dynamics.

### Automated analysis of Ca^2+^ imaging data

**Figure 2:**
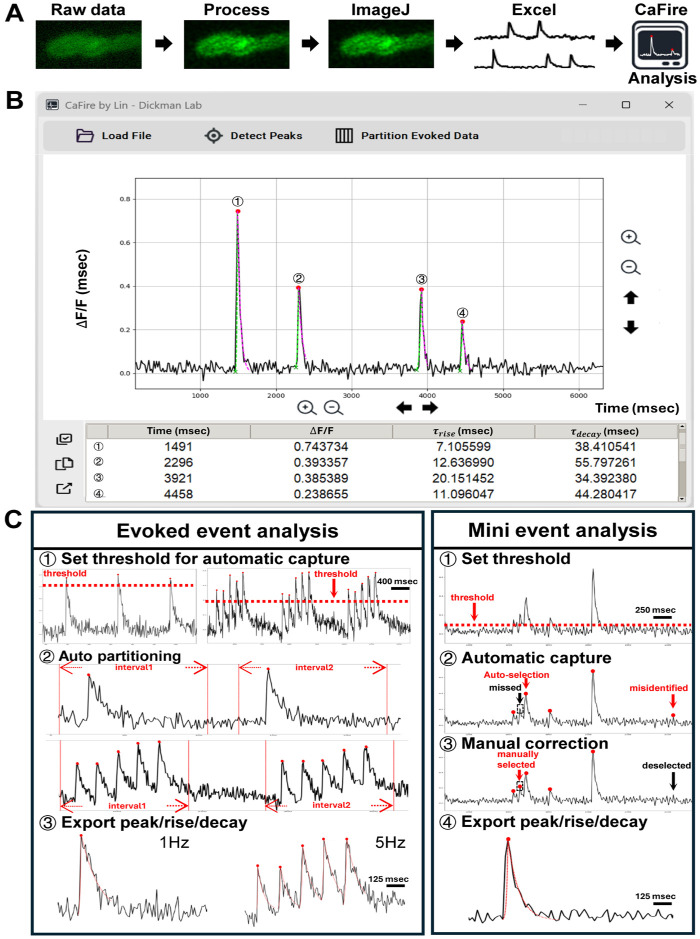
“CaFire” - a Python-based analysis program for quantifying synaptic Ca^2+^ imaging data. **(A)** Workflow showing how Ca^2+^ imaging data and downstream analysis is performed. Raw timelapse movies are processed with SVI Huygens software to correct and deconvolve image artifacts. ROIs are then selected in ImageJ Fiji software and intensities are extracted. Data are exported to Excel for manual inspection or directly analyzed using CaFire software. **(B)** Screenshot of the CaFire user interface. Users can load fluorescence intensity data, detect Ca^2+^ events, partition evoked events, calculate peak amplitude, and rise and decay time constants. Detected events are automatically marked on the raw traces and associated values are displayed in the data table below. **(C)** Examples of two distinct analysis pipelines implemented in CaFire. *Evoked event analysis*: (1) Thresholds are set for automatic peak detection; (2) Events are automatically partitioned based on stimulus intervals (e.g., 1 Hz and 5 Hz); (3) Parameters such as peak amplitude, rise time constant (⊤_rise_), and decay time constant (⊤_decay_) are calculated using exponential fits. *Mini event analysis*: (1) Users define amplitude thresholds for event detection; (2) CaFire automatically identifies candidate events; (3) Missed or misidentified events can be manually corrected; (4) Event parameters are exported for each validated event.

Current approaches to analyze synaptic Ca^2+^ imaging data either repurpose software designed to analyze electrophysiological data or use custom software developed by groups for their own specific needs. We therefore developed a new Python-based software platform capable of automated detection and quantification of synaptic Ca^2+^ events. This program, which we named “CaFire”, analyzes acquired imaging data followed by optional processing such as deconvolution (Fig. 2A). The Ca^2+^ imaging data are then imported into ImageJ to select Regions of Interest (ROIs), which are finally exported to a graphical program such as Microsoft Excel. The CaFire software reads the Excel file and performs all downstream analysis.

CaFire can analyze both presynaptic evoked and postsynaptic quantal Ca^2+^ events with a user-friendly graphical interface that enables parameter adjustment and event visualization (Fig. 2B). For both quantal and evoked events, peak detection is based on customizable parameters, including threshold amplitude, peak width, and minimum inter-peak distance. These parameters can be adjusted to optimize detection sensitivity and specificity, while misidentified events can be manually corrected. Once events are identified, CaFire then quantifies key parameters, such as peak amplitude (ΔF/F), and rise time constants based on an exponential growth formula, and decay time constants using a natural logarithmic decay formula (Fig. 2C; see Methods). In addition, evoked Ca^2+^ events can be partitioned at different stimulation frequencies (e.g., 1 Hz, 5 Hz) to assess frequency-dependent dynamics. All quantification results are displayed in real-time and can be exported in a tabular format for downstream statistical analysis. This automated workflow significantly reduces analysis time and user variability in Ca^2+^ imaging data analysis when compared to a variety of other platforms we have tried, providing uniform, accurate, and reproducible measurements. CaFire’s ability to analyze both evoked and quantal events makes it a versatile tool for quantifying synaptic Ca^2+^ imaging data.

### Benchmarking presynaptic Ca^2+^ sensors

**Figure 3:**
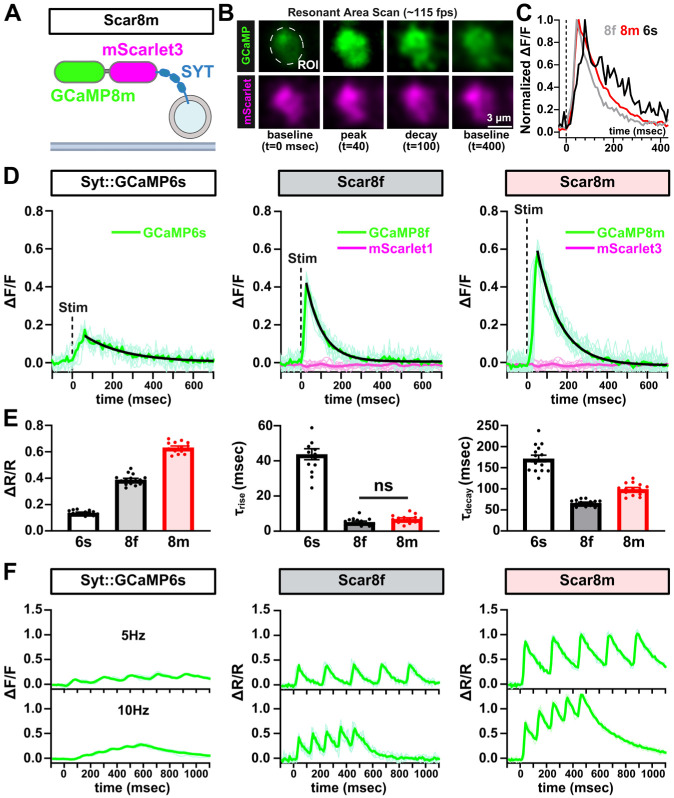
Scar8m is an optimal presynaptic Ca^2+^ indicator. **(A)** Schematic of the UAS-Syt::mScarlet3::GCaMP8m (Scar8m) ratiometric Ca^2+^ indicator consisting of mScarlet3 and GCaMP8m targeted to synaptic vesicles via fusion to Synaptotagmin (SYT). **(B)** Representative images of a MN-Ib bouton expressing Scar8m (*w*;*OK319-GAL4/+*;*Scar8m/+*) resonant scanned at ~115 fps. Fluorescence from GCaMP8m (green) and mScarlet3 (magenta) is shown at baseline, peak, decay, and recovery to baseline. **(C)** Normalized Ca^2+^ signals following single action potential (AP) stimulation. Averaged traces compare the kinetics of GCaMP6s, GCaMP8f, and GCaMP8m. **(D)** Representative GCaMP and mScarlet signals recorded from Syt::GCaMP6s, Scar8f, and Scar8m in response to single AP stimuli. The mScarlet reference signal remains stable throughout the recording. **(E)** Quantification of average peak amplitude (ΔR/R, GCaMP/mScarlet ratios), rise time constant (⊤_rise_), and decay time constant (⊤_decay_) from the indicated sensors. Scar8m yields significantly higher peak ΔR/R signals, similar rise time kinetics, and a modestly slower decay compared to Scar8f. **(F)** Presynaptic Ca^2+^ responses of the indicated sensors to 5 Hz and 10 Hz stimulation trains. All comparisons in bar graphs are statistically significant unless explicitly noted otherwise. Error bars represent ±SEM. Unless indicated by an “ns” label, all values are significantly different; detailed statistics including p-values are presented in Table S1.

To evaluate the performance of presynaptically targeted Ca^2+^ indicators, we first compared the responses of SYT::GCaMP6s, Scar8f, and Scar8m at motor neuron Ib (MN-Ib) terminals. Live confocal imaging confirmed robust expression of all sensors at presynaptic boutons, with colocalized GCaMP and mScarlet fluorescence (Fig. 3A,B). Ca^2+^ transients were elicited by single action potential (AP) electrical stimulation, and fluorescence responses were recorded and analyzed (Fig. 3C,D). Scar8m produced the highest amplitudes of GCaMP/mScarlet (ΔR/R) ratios, with GCaMP8m showing 345.7% higher SNR over GCaMP6s, and 55.7% higher compared to GCaMP8f (Fig. 3D,E). Both Scar8f and Scar8m clearly outperformed GCaMP6s in both sensitivity and speed (Fig. 3D,E), as expected. Importantly, while GCaMP8f exhibited slightly faster decay time constants (66.6 msec vs 99.2 msec), rise time constants were not significantly different between the two sensors (5.3 msec vs 7.0 msec) (Fig. 3E). Thus, Scar8m exhibited an optimal balance between kinetics and signal strength.

To assess sensor performance during repetitive activity, we tested responses to 5 stimuli at 5 Hz and 10 Hz stimulation trains. While responses were muted using SYT::GCaMP6s, Scar8f and Scar8m consistently demonstrated robust responses and separation across repetitive stimulation (Fig. 3F). Together, we conclude that because of the superior sensitivity, ratiometric properties, and fast kinetics, Scar8m is an optimal GECI to quantify stimulated Ca^2+^ responses from presynaptic terminals.

**Figure 4:**
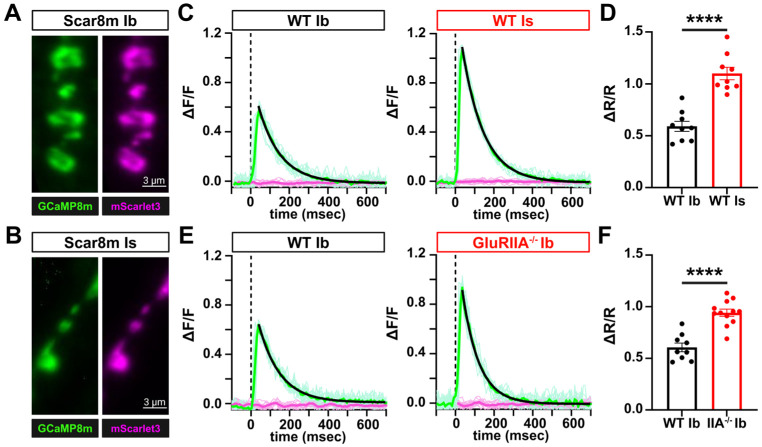
Scar8m captures differences in Ca^2+^ levels between motor neuron subtypes and after plasticity. **(A,B)** Representative images of Scar8m expressed at both MN-Ib (A) and MN-Is (B) motor neuron subtypes immunostained with anti-GFP. **(C)** ΔF/F traces of GCaMP8m and mScarlet3 responses from single AP stimulation at MN-Ib and MN-Is, with ~2x higher responses observed at MN-Is over -Ib, as expected. **(D)** Quantification of ΔR/R responses from the two inputs. **(E)** ΔF/F traces of GCaMP8m and mScarlet3 responses from single AP stimulation at MN-Ib in wild type (*w;OK319-GAL4/+;Scar8m/+*) and *GluRIIA* mutants (*w;OK319-GAL4,GluRIIA*^*PV3*^*/GluRIIA*^*PV3*^;*Scar8m/+*), which express presynaptic homeostatic plasticity (PHP). Note the enhanced presynaptic Ca^2+^ levels induced after PHP plasticity. **(F)** Quantification of ΔR/R responses from the indicated genotypes. Error bars represent ±SEM. Detailed statistics including p-values are provided in Table S1.

To determine whether the sensitivity of Scar8m is sufficient to quantitatively report biologically relevant differences in evoked presynaptic Ca^2+^ responses, we first compared evoked responses between MN-Ib and MN-Is terminals. Previous studies using chemical indicators have shown MN-Is exhibits ~2-3x increased Ca^2+^ responses over MN-Ib (Lu et al., 2016, He et al., 2023). First, we confirmed robust Scar8m expression in both MN-Ib and -Is using the OK319-Gal4 driver (Fig. 4A,B). Next, Scar8m responses to single AP stimulation revealed a >two-fold increase at MN-Is terminals compared to MN-Ib (Fig. 4C,D). Next, we assessed changes in presynaptic Ca^2+^ responses at MN-Ib in wild type and *GluRIIA* mutants, in which a process called presynaptic homeostatic potentiation (PHP) is known to be induced (He et al., 2025, Davis et al., 2015). In PHP, diminished postsynaptic GluR functionality is offset through a homeostatic signaling system which enhances neurotransmitter release (Goel et al., 2021, Delvendahl et al., 2019). PHP induces an increase in presynaptic Ca^2+^ influx to promote additional synaptic vesicle release (Müller et al., 2012, Chien et al., 2025), where chemical indicators such as Oregon Green Bapta-1 (OGB-1) have shown ~30% increase Ca^2+^ levels after PHP (Müller et al., 2012). Importantly, Scar8m responses in *GluRIIA* mutant MN-Ib boutons showed significantly elevated ΔR/R amplitudes compared to wild type, with a ~53% enhancement (Fig. 4E,F). Thus, Scar8m can resolve biologically relevant differences in presynaptic Ca^2+^ with high sensitivity, on par with what chemical indicators have shown.

### Evaluating the Bar8f sensor to resolve local active zone Ca^2+^ signals

**Figure 5:**
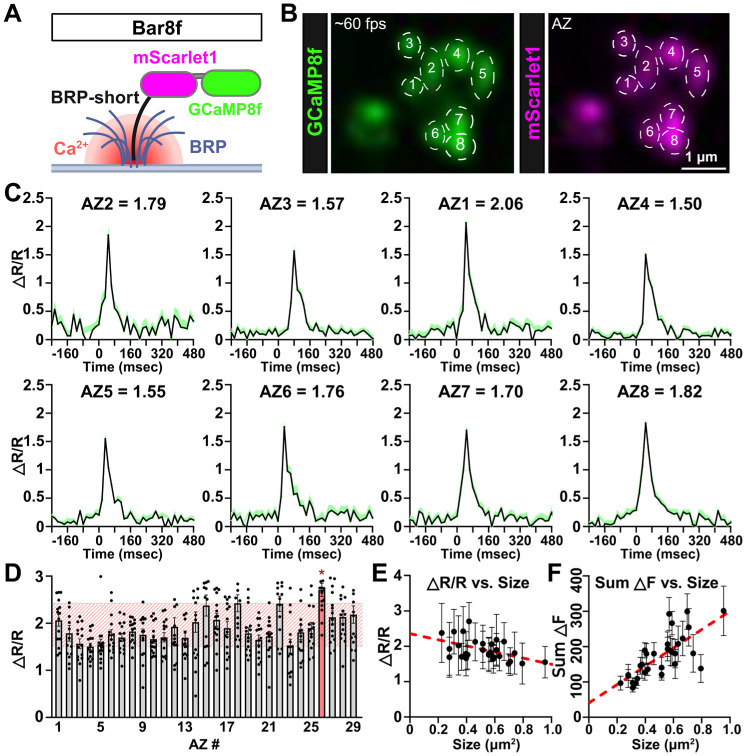
Evaluating the ability of Bar8f to capture active zone-specific Ca^2+^ dynamics. **(A)** Schematic of the Bar8f indicator, consisting of GCaMP8f and mScarletl fused to the BRP-short domain, which traffics to individual active zones (AZs). **(B)** Live confocal image of a single MN-Ib bouton expressing Bar8f (*w*;*OK319-GAL4/+;Bar8f/+*). GCaMP8f (green) and mScarlet1 (magenta) localize to AZ puncta. Eight individual AZs are shown as ROIs. **(C)** Representative ΔR/R traces of single AP-evoked Ca^2+^ responses at the eight annotated AZs shown in (B). Numerical values indicate peak ΔR/R amplitude for each AZ. **(D)** Bar graph showing peak ΔR/R values from 29 individual AZs collected across multiple boutons from different NMJs. Note that only one AZ shows Ca^2+^ responses that are significantly different from the others (highlighted in red). **(E)** Scatter plots relating AZ size (measured by mScarletl fluorescence area) to ΔR/R. A weak negative correlation is observed between ΔR/R and AZ size, likely due to overestimation of ROI boundaries during manual AZ segmentation. **(F)** Scatter plot showing summed ΔF responses as a function of AZ size. A strong positive correlation is observed between summed ΔF and AZ size, reflecting the expected scaling of total GCaMP fluorescence signals with abundance at larger AZs. Error bars indicate ±SEM. Additional statistical details are shown in Table S1.

Differences in Ca^2+^ influx and buffering at individual release sites contribute to functional differences at synapses (Dittman et al., 2019, Jackman et al., 2017, McCarthy et al., 2024). While Scar8m is an excellent sensor to define spatially-averaged presynaptic Ca^2+^ levels at individual boutons, the ability to resolve the heterogeneous Ca^2+^ signals within individual boutons during neurotransmission would be a powerful tool to interrogate presynaptic function. Thus, we engineered the Bar8f sensor to localize a ratiometric GCaMP8f/mScarlet cassette to AZs by fusion to the BRP-short motif (Fig. 5A). While some previous studies have suggested a similar approach can resolve AZ-specific differences in Ca^2+^ influx (Akbergenova et al., 2018), from the outset we were cognizant of many challenges to this line of investigation. First, theoretical calculations suggest that Ca^2+^ diffuses and equilibrates at a rate of <3 msec across an entire bouton, faster than most sensors would respond before receiving Ca^2+^ contributions from other release sites (Maravall et al., 2000, Sinha et al., 1997, Helmchen et al., 1997). Second, it is difficult to resolve individual AZs from live imaging of boutons, as many are closely spaced when flattened 2D images are taken. Finally, at the Drosophila NMJ, individual AZ labeled by BRP are very small, with diameters averaging ~300 nm (Ramesh et al., 2021). To spatially resolve these discrete AZs during Ca^2+^ imaging, high spatial resolution is required, which often necessitates reducing the scanning rate. Furthermore, when employing dual-channel imaging to simultaneously capture signals from GCaMP and mScarlet, sequential scanning is typically implemented to minimize spectral crosstalk between channels. These technical constraints collectively limit the achievable frame rate for AZ-level Ca^2+^ imaging to approximately 60 frames per second (fps) using resonant area scanning (Chen et al., 2024), although faster frame rates can be achieved with CMOS cameras.

Despite these acknowledged challenges and potential confounds, we sought to test the ability of Bar8f to resolve local AZ Ca^2+^ dynamics following single AP stimulation. Individual AZs were identified at MN-Ib boutons using basal mScarlet fluorescence, then evoked Ca^2+^ signals were measured using resonant area scanning (Fig. 5B). Peak ΔR/R values were measured at individual AZs (Fig. 5B,C), where typical ΔR/R values at individual AZs ranged from ~1.5 - 2.5, with only a small number (3-4%) being significantly different from this range (Fig. 5D). These results, while taking into account the caveats detailed above, are consistent with the theoretical estimates of Ca^2+^ levels being equilibrated across all AZs before the Ca^2+^ indicator can capture local differences.

To further assess Ca^2+^ levels at individual AZ captured by the Bar8f sensor, we analyzed our imaging data in more detail. First, we plotted AZ ΔR/R values as a function of AZ size, defined by the mScarlet mean intensity signal. This plot suggested a slight negative correlation (Fig. 5E), which may be due to an overestimation of ROI area during manual segmentation. Indeed, larger ROIs drawn around larger AZs might lead to the inclusion of surrounding low-signal pixels to ultimately dilute the ratiometric peak GCaMP response and lead to an inaccurate reduction in the measured ΔR/R signal at larger AZs. Second, previous studies have clearly shown that larger AZs contain a higher abundance of Ca_v_2 channels (Akbergenova et al., 2018, He et al., 2023, Medeiros et al., 2024), so larger AZs should have an increase in the absolute value of Ca^2+^ influx following stimulation. We therefore plotted the summed integrated fluorescence signal (sum ΔF) as a function of AZ area. As expected, summed ΔF values scaled strongly and positively with AZ size (Fig. 5F), reflecting a greater number of Ca_v_2 channels at larger AZs. It is important to note that we cannot rule out more subtle differences in ΔR/R variation due to the limited resonant area scan speeds of our imaging system. Nonetheless, these findings suggest that Bar8f can report total Ca^2+^ levels at individual AZs. However, we interpret the observed relative consistency of ΔR/R at individual AZs as indicative of GCaMP-based sensors, targeted to individual AZs, being unable to reliably resolve local Ca^2+^ changes before equilibration within an individual bouton.

### SynapGCaMP8 sensors resolve quantal events at postsynaptic compartments

Finally, we evaluated the performance of SynapGCaMP6f, SynapGCaMP8f, and SynapGCaMP8m sensors to monitor postsynaptic “quantal” events at the Drosophila NMJ. These quantal events reflect the rapid ionic influxes that result from the spontaneous release of single synaptic vesicles, which open postsynaptic GluRs and allow passage of Na^+^ and Ca^2+^ ions (Grienberger et al., 2012). Electrophysiological methods are the gold standard to report these mEPSP events, and we were particularly interested in determining whether SynapGCaMP variants are capable of detecting quantal events with similar sensitivity.

**Figure 6:**
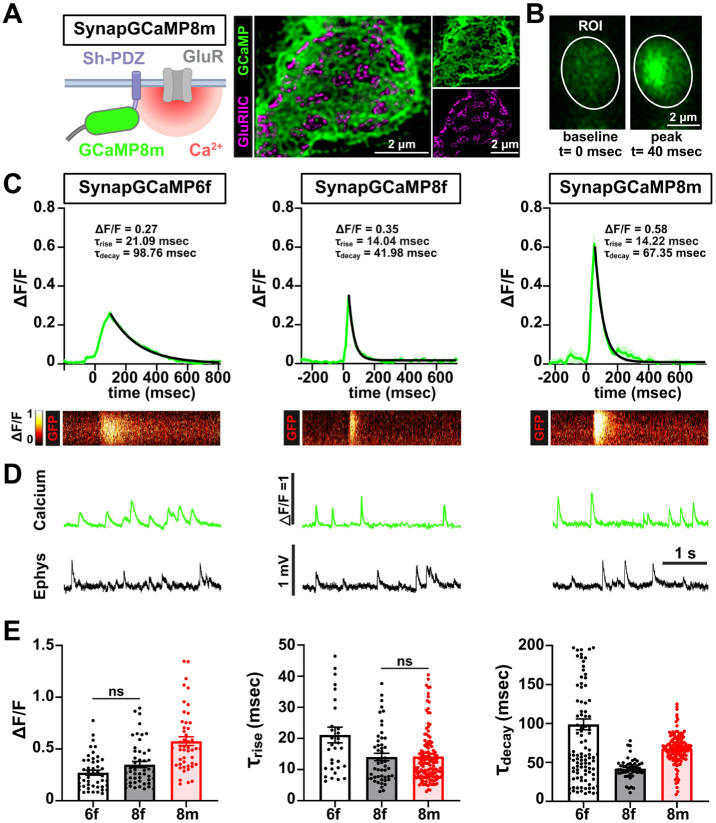
SynapGCaMP8m is an optimal postsynaptic Ca^2+^ indicator. **(A)** Schematic of the SynapGCaMP8m reporter, with the GCaMP indicator targeted to postsynaptic compartments near glutamate receptors via a Shaker-PDZ motif. Super resolution image using STED microscopy showing the GCaMP8m reporter is localized outside of GluRs. **(B)** Live confocal images of muscle 6 NMJ boutons expressing SynapGCaMP8m were performed using resonant area scans. The indicated ROI shows representative frames at baseline and peak quantal Ca^2+^ transients acquired at ~115 fps. **(C)** Averaged single miniature Ca^2+^ events recorded from SynapGCaMP6f, −8f, and −8m. Traces show ΔF/F responses with fitted rise and decay time constants (⊤), along with amplitude values for each indicator. SynapGCaMP8m yields the highest peak signal and maintains rapid kinetics. The corresponding heatmaps below show the spatiotemporal fluorescence dynamics of each indicator. **(D)** Representative traces of quantal events imaged with the indicated SynapGCaMP sensor and quantal events (mEPSPs) recorded using electrophysiology. **(E)** Quantification of ΔF/F peak amplitude, and rise and decay time constants (⊤_rise_ and ⊤_decay_) for each SynapGCaMP variant. All comparisons in bar graphs are statistically significant unless “ns” is shown. Error bars indicate ±SEM. Additional statistical details, including p-values, are presented in Table S1.

We first confirmed that all SynapGCaMP variants expressed well and trafficked to postsynaptic compartments, localizing with the scaffold DLG while encompassing but being distinct from glutamate receptive fields, which is particularly apparent with super resolution STED microscopy (Fig. 6A). Using resonant area scanning at ~115 fps, we imaged and analyzed single quantal Ca^2+^ events for each SynapGCaMP variant. Averaged traces revealed a progressive improvement in response kinetics and sensitivity across the indicator series (Fig. 6B,C). SynapGCaMP6f exhibited modest sensitivity, with ΔF/F values of 0.27 and relatively slow kinetics (⊤_rise_=21 msec, ⊤_decay_=99 msec), while SynapGCaMP8f achieved faster responses and higher sensitivity (ΔF/F=0.35; ⊤_rise_=14 msec, ⊤_decay_=42 msec). SynapGCaMP8m exhibited the highest peak amplitude (ΔF/F=0.58), with similarly fast rise times (⊤_rise_=14 msec) and moderate slowing of the decay (⊤_decay_=67 msec), striking an optimal balance between speed and sensitivity (Fig. 6C,E). SynapGCaMP quantal signals appeared to qualitatively reflect the same events measured with electrophysiological recordings (Fig. 6D). We conclude that SynapGCaMP8m is an optimal indicator to measure quantal transmission events at the synapse.

**Figure 7:**
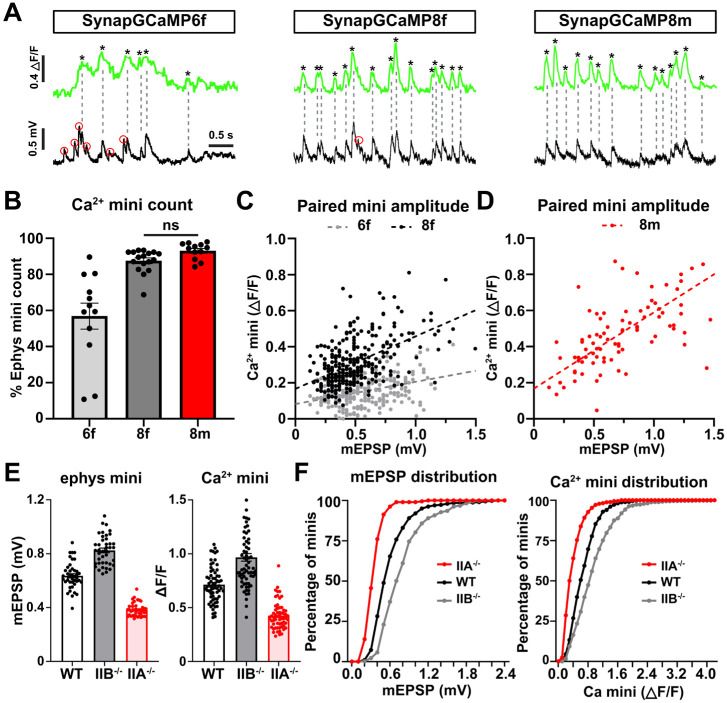
SynapGCaMP8m resolution approaches that of electrophysiology. **(A)** Simultaneous recordings of quantal events at MN-Ib boutons using the indicated SynapGCaMP variant (green) and electrophysiology (black) after silencing MN-Is (*w;Is-GAL4/+;UAS-BoNT-C/SynapGCaMP*). Red circles indicate mEPSP events not captured by SynapGCaMP. SynapGCaMP8m captures quantal events with high sensitivity, comparable to electrophysiology. **(B)** Quantification of the proportion of quantal events captured by the indicated SynapGCaMP variant as a proportion of the total mEPSP events recorded by electrophysiology. SynapGCaMP6f detects only about half of electrophysiological events, while both SynapGCaMP8f and SynapGCaMP8m capture nearly all mEPSPs. **(C)** Scatter plot of paired miniature event amplitudes recorded simultaneously by SynapGCaMP6f and −8f and electrophysiology. **(D)** Scatter plot of paired miniature event amplitudes recorded simultaneously by SynapGCaMP8m and electrophysiology. A linear relationship with high correlation is observed for SynapGCaMP8m, indicating that optical signals scale proportionally with quantal amplitude. **(E)** Bar plots showing average mEPSP amplitudes (left) and ΔF/F amplitudes of quantal Ca^2+^ events (right) in the indicated genotypes (same as (A) above but with *GluRIIA* or *GluRIIB* mutant alleles included). SynapGCaMP8m accurately resolves quantal size differences with similar resolution as the electrophysiological data, with quantal amplitudes in both datasets exhibiting the expected differences (GluRIIA^−/−^<WT<GluRIIB^−/−^). **(F)** Cumulative frequency distributions of mEPSP amplitudes (left) and Ca^2+^ mini event amplitudes (ΔF/F) (right); each are significantly different using the Kolmogorov-Smirnov Test. See Table S1 for full statistical details including p-values.

Next, we systematically assessed the ability of the SynapGCaMP variants to resolve quantal events. In particular, we switched to a widefield Ca^2+^ imaging system mounted on an electrophysiology rig so that we could perform simultaneous Ca^2+^ imaging and electrophysiological recordings of mEPSP events. To simplify our analysis, we isolated miniature events from MN-Ib inputs by silencing MN-Is transmission using selective expression of BoNT-C (Han et al., 2022) (see Methods). Sharp electrode intracellular recordings and Ca^2+^ imaging from muscle 6 MN-Ib boutons expressing SynapGCaMP6f, −8f, or −8m revealed increasing fidelity of quantal events (Fig. 7A,B). Matched GCaMP and mEPSP recordings showed a high degree of temporal correspondence and sensitivity between mEPSPs and Ca^2+^ transients, with SynapGCaMP8m capturing the vast majority of electrophysiologically detected mEPSP events (Fig. 7A,B). Indeed, mEPSP events that failed to register a detectable Ca^2+^ transient were most frequent with SynapGCaMP6f, rare with SynapGCaMP8f, and nearly absent with SynapGCaMP8m. Across samples, SynapGCaMP8f and SynapGCaMP8m detected nearly all electrophysiologically recorded mEPSPs, with detection rates of 88% and 93% respectively, whereas SynapGCaMP6f detected only 57% of these events. (Fig. 7B). Importantly, when quantal amplitudes from paired mEPSPs and corresponding Ca^2+^ transients were plotted, SynapGCaMP8m exhibited a strong linear correlation with electrophysiological recordings (Pearson’s r =0.810, R^2^ =0.656; p<0.0001, n=216), demonstrating that the indicator reliably tracks quantal variability (Fig. 7D). SynapGCaMP6f and SynapGCaMP8f also showed significant positive correlations, although weaker than SynapGCaMP8m (6f: r =0.458, R^2^=0.210, n=283; 8f: r =0.732, R^2^ =0.537, n=298; all p<0.0001) (Fig. 7C). Thus, SynapGCaMP8m provides a sensitive indicator of quantal events, approaching the sensitivity of electrophysiological recordings.

In our final set of experiments, we focused on benchmarking the ability of SynapGCaMP8m to resolve physiologically relevant differences in quantal events compared to electrophysiology. Specifically, we compared electrophysiological recordings and SynapGCaMP8m imaging of quantal events in three genotypes with known differences in quantal amplitudes: wild type as the baseline control, *GluRIIA* mutants with diminished quantal amplitudes, and *GluRIIB* mutants with enlarged quantal events (Han et al., 2023). Electrophysiological recordings from each of the three genotypes confirmed the expected differences, with baseline mEPSPs from MN-Ib averaging ~0.64 mV, *GluRIIB* mutants ~0.82 mV, and *GluRIIA* mutants ~0.38 mV (Fig. 7E), as shown in previous studies (Han et al., 2023, Han et al., 2022, He et al., 2023). Plotting the mEPSP distribution as a cumulative probability histogram also showed the expected distribution of these genotypes (Fig. 7F). We next performed a similar analysis of quantal events imaged with SynapGCaMP8m. These results demonstrated near identical differences in average quantal amplitude and distribution in quantal events imaged from wild type, *GluRIIA,* and *GluRIIB* mutants (Fig. 7E,F). These data demonstrate that SynapGCaMP8m can capture physiologically relevant differences in quantal events with similar sensitivity as electrophysiology.

## DISCUSSION

This study presents a significant advancement in our capacity to optically interrogate Ca^2+^ dynamics at synaptic compartments of the *Drosophila* neuromuscular junction, approaches that in principle can be extended to other systems. By systematically engineering and rigorously benchmarking a new suite of ratiometric GECIs – presynaptic Scar8m, active-zone targeted Bar8f, and postsynaptic SynapGCaMP8m – and complemented with the development of the CaFire automated analysis platform, we have overcome limitations of previous tools. These indicators provide a powerful toolkit for dissecting the Ca^2+^ signals that mediate and control synaptic transmission and plasticity, moving us closer to all optical interrogation with similar resolution as electrophysiology. These results demonstrate that careful sensor design, incorporating the latest GCaMP8m variant and optimized ratiometric partners like mScarlet3, can yield probes with unprecedented performance in this model system.

Presynaptic Ca^2+^ imaging with Scar8m not only surpasses previous GECIs but also rivals, and in some respects exceeds, the capabilities of traditional synthetic Ca^2+^ indicators. While chemical dyes offer rapid kinetics (Hendel et al., 2008, Lock et al., 2015, Macleod, 2012, Lnenicka et al., 2006, Justs et al., 2022, Lu et al., 2016), their utility is often hampered by inconsistent loading/concentrations, lack of cell-type specificity, and challenges in ratiometric quantification within the confined space of presynaptic boutons. Indeed, traditional approaches to load dyes into fly motor neurons relies on cutting the nerve while bathed in solution, and waiting for the dye to diffuse the considerable distance to presynaptic terminals, where variation in dye concentration can confound analyses (McMahon et al., 2018, Macleod, 2012). Scar8m, targeted to synaptic vesicle pools at boutons via fusion to Synaptotagmin along with the ratiometric mScarlet3 motif, circumvents these issues, providing robust, genetically targeted, and stoichiometric and quantifiable Ca^2+^ measurements, spatially-averaged and confined to individual boutons. Further, the superior sensitivity and relatively fast kinetics of Scar8m enabled the resolution of physiologically meaningful differences in presynaptic Ca^2+^ levels, such as those between MN-Ib and MN-Is terminals and the elevations associated with presynaptic homeostatic plasticity. When compared to previous studies that used chemical dyes including OGB-1, rhod, and Fura (Lu et al., 2016, He et al., 2023, Müller et al., 2012), the performance of Scar8m was in line with or exceeded these indicators. Specifically, GCaMP8m exhibits a higher SNR compared to both OGB-1 and Fura-2. Notably, Scar8m achieves a ΔF/F of approximately 0.63, a level comparable to that of OGB-1 (He et al., 2023, Müller et al., 2012). Although Scar8m’s decay time constant is moderately slower compared to chemical dyes (~100 msec vs. ~60 msec for OGB-1 and ~40 msec for Rhod) (Macleod et al., 2002, Justs et al., 2022), the performance of Scar8m is on par with the rise kinetics of chemical dyes in many cases, with a single-AP rise time of ~12 msec compared to ~8 msec for Fura-2 and <2 msec for OGB-1 (Maravall et al., 2000, Sinha et al., 1997). It is important to note that with our scan speeds of ~115 fps, our estimate of rise times are likely slowed. This capacity to faithfully report presynaptic Ca^2+^ changes, previously accessible primarily via chemical dyes, underscores Scar8m’s power as a tool for investigating presynaptic function with enhanced precision and reliability.

Equally significant is the performance of the postsynaptically targeted SynapGCaMP8m, which we have shown achieves a sensitivity for detecting quantal Ca^2+^ events that approaches the gold standard of electrophysiological recordings. Previous optical methods often struggled to reliably capture these small, stochastic events, particularly in the case of glutamate receptor loss or perturbation, and have never previously been benchmarked simultaneously with electrophysiological recordings to determine their fidelity (Newman et al., 2017, Peled et al., 2014, Han et al., 2022). We found that SynapGCaMP6f failed to capture over 40% of mEPSP events, while the GCaMP8m version reliably detected over 90% of these events, even in cases of diminished amplitude due to *GluRIIA* loss. Leveraging the high responsivity of GCaMP8m enabled substantially improved peak amplitudes and kinetics. Furthermore, the strong correlation observed between the amplitudes of optical quantal events and their corresponding mEPSPs, along with the ability to accurately resolve known differences in quantal size in *GluRIIA* and *GluRIIB* mutants, serves to validate SynapGCaMP8m as a high-fidelity reporter of postsynaptic activity. This opens exciting avenues for all-optical interrogation of quantal parameters, synaptic strength, and changes due to plasticity with subcellular spatial resolution.

Our investigation into attempting to capture local Ca^2+^ signals using an active zone-targeted, ratiometric GCaMP8f based indicator, Bar8f, while ambitious, also yielded important insights despite the inherent technical challenges. Bar8f was able to sensitively report Ca^2+^ changes at individual AZs following single AP electrical stimulation, as evidenced by the positive scaling of summed fluorescence with AZ size, as expected by variation in Cav2 channel abundance shown in previous studies(Medeiros et al., 2024, Cunningham et al., 2022, Gratz et al., 2019). However, the ratiometric intensity signals showed relative consistency across AZs following single AP stimulation. This finding, coupled with theoretical considerations of Ca^2+^ diffusion rates (Sinha et al., 1997, Helmchen et al., 1997), suggests that within the temporal resolution of current GCaMP sensors, and likely synthetic dyes, Ca^2+^ concentrations are likely to equilibrate across AZs within an individual bouton before distinct local concentration differences can be reliably resolved. There are a number of other potential confounds precluding a definitive conclusion about the ability of Bar8f to resolve local Ca^2+^ changes, including challenges in resolving individual AZs and imaging speeds. These observations are critical for interpreting data acquired from AZ-targeted GECIs and guide future efforts to capture localized Ca^2+^ nanodomain dynamics, likely requiring sensors with even faster kinetics and/or alternative imaging modalities.

The suite of next-generation GECIs, particularly Scar8m and SynapGCaMP8m, coupled with the CaFire analysis pipeline, represents a substantial toolkit for studying synaptic Ca2+ signaling. At the Drosophila NMJ, these tools provide researchers with unprecedented optical access to both presynaptic Ca^2+^ influx driving neurotransmitter release and postsynaptic Ca2+ transients reflecting quantal events, with performance characteristics that rival traditional, more invasive methods. The ability to perform ratiometric, genetically-targeted imaging with such high sensitivity and temporal resolution will help to accelerate discoveries into the molecular mechanisms of synaptic transmission, plasticity, and disease etiology. More generally, the strategies used here in the fly system can inspire similar approaches to be employed in other systems. Future studies leveraging these advanced sensors can now tackle complex questions regarding the spatial and temporal dynamics of Ca^2+^ signaling within synaptic compartments, the modulation of these signals during various forms of plasticity, and their dysregulation in models of neurological disorders, further cementing the Drosophila NMJ as a premier system for fundamental synaptic research.

## MATERIALS AND METHODS

### Fly stocks:

Drosophila stocks were raised at 25°C using standard molasses food. Unless otherwise specified, the *w*^*1118*^ strain was used as the wild-type control as this is the genetic background in which all genotypes were bred. All experiments were performed on Drosophila third-instar larvae of both sexes unless otherwise noted. See Table S2 (Key Resources Table) for a full list of all fly stocks and their sources used in this study.

### Molecular biology:

To generate the transgenic constructs used in this study, we built upon previously established tools. First, SynapGCaMP8m was engineered by modifying the previous SynapGCaMP8f construct (Han et al., 2022), which was in turn based on the original SynapGCaMP6f construct (Newman et al., 2017). Gibson assembly reactions were used to convert the few relevant amino acid differences between GCaMP8f and GCaMP8m (Zhang et al., 2023). Similarly, UAS-Syt::mScarlet3::GCaMP8m (Scar8m) was generated using similar approaches to modify the previously described UAS-Syt::mScarlet::GCaMP8f plasmid (Li et al., 2021), changing both the GCaMP8f and mScarlet sequences to GCaMP8m and mScarlet3 (Gadella et al., 2023). Finally, UAS-BrpS::mScarlet::GCaMP8f (Bar8f) was engineered based on the previous UAS-BrpS::mCherry::GCaMP6s transgene (Kiragasi et al., 2017), where the GCaMP6s sequence was replaced with GCaMP8f, and the mCherry tag was substituted with mScarlet. SynapGCaMP transgenes were inserted randomly through p-element transposition, while all other transgenes were inserted into the attP40 (II) or VK27 (III) sites by BestGene Inc. (Chino Hills, CA). All constructs were verified by Sanger sequencing prior to injection, and transgenic fly stocks were established and maintained under standard laboratory conditions.

### Immunocytochemistry:

Third-instar larvae were dissected in ice cold 0 Ca^2+^ HL-3 and immunostained as described (Li et al., 2021, Han et al., 2023). Briefly, larvae were either fixed in 100% ice-cold methanol for 5 mins or PFA for 10 mins followed by washing with PBS containing 0.1% Triton X-100 (PBST). Samples were blocked with 5% Normal Donkey Serum for 1 hour and incubated with primary antibodies overnight at 4°C. Preparations were washed for 10 mins thrice in PBST, incubated with secondary antibodies for 2 hours at room temperature, washed thrice again in PBST, and equilibrated in 70% glycerol. Prior to imaging, samples were transferred in VectaShield (Vector Laboratories, Burlingame, CA) and mounted on glass cover slides. Details of all antibodies and sources are listed in Table S2.

### Electrophysiology:

All dissections and electrophysiological recordings were performed as described (Kikuma et al., 2019) in modified hemolymph-like saline (HL-3) containing (in mM): 70 NaCl, 5 KCl, 10 MgCl_2_, 10 NaHCO_3_, 115 Sucrose, 5 Trehelose, 5 HEPES, pH=7.2, and CaCl_2_ at 0.4 mM unless otherwise specified. Internal guts, brain and the ventral nerve cord were subsequently removed to achieve fully dissected preparations. Recordings were carried out on an Olympus BX61 WI microscope stage equipped with a 40x/0.8 NA water-dipping objective and acquired using an Axoclamp 900A amplifier (Molecular Devices). All recordings were conducted on abdominal muscle 6, segment A3 of third-instar larvae of both sexes. Data were acquired from cells with an initial resting potential between −60 and −80 mV, and input resistances >5 MΩ. Miniature excitatory postsynaptic potentials (mEPSPs) were recorded without any stimulation and low pass filtered at 1 kHz. The mEPSPs for each sample were recorded for 60 secs, analyzed with MiniAnalysis (Synaptosoft), and the average mEPSP amplitude for each NMJ was calculated. Excitatory postsynaptic potentials (EPSPs) were recorded by delivering 20 electrical stimuli at 0.5 Hz with 0.5 msec duration to motor neurons using an ISO-Flex stimulus isolator (A.M.P.I.) with stimulus intensities set to avoid eliciting multiple EPSPs.

### Confocal imaging:

Dissections and live Ca^2+^ imaging was performed as described (Chien et al., 2025, Chen et al., 2024) on muscle 6 NMJs. Wandering third-instar larvae were dissected and imaged in 1.8 mM Ca^2+^ HL3 saline as described above. Ca^2+^ imaging was conducted using a Nikon A1R resonant scanning confocal microscope equipped with a 60x/1.0 NA water-immersion objective. GCaMP was excited with 488 nm and mScarlet/mCherry with 561 nm lasers. ROIs focused on terminal boutons of MN-Ib or -Is motor neurons. Resonant area scans of synaptic boutons were acquired at 115 fps with a scanning area of 256x32 or 256x64 pixels, using a zoom factor of 8x. Presynaptic Ca^2+^ imaging was conducted for 15 secs per acquisition while delivering electrical stimulation at 1 Hz (1 msec pulse duration) or at 5 Hz and 10 Hz (five pulses per 2-second burst). To prevent muscle contraction during imaging, 7 mM L-Glutamic acid monosodium salt (Sigma-Aldrich, Cat# G5889) was added to the HL3 saline. In contrast, postsynaptic Ca^2+^ imaging was performed for 60 secs without electrical stimulation, as only quantal events were monitored. Data was collected from at least three biological replicates per genotype.

Confocal images of fixed tissue were acquired with a 100x APO 1.45NA oil immersion objective using separate channels with four laser lines (405 nm, 488 nm, 561 nm, and 647 nm) as described (Perry et al., 2017). Z-stacks were obtained on the same day using identical gain and laser power settings with z-axis spacing between 0.13 and 0.2 μm and pixel size of 0.06 μm for all samples within an individual experiment, from at least 8 NMJs acquired from at least four different animals. Raw confocal images were deconvolved with SVI Huygens Essential 22.10 using built-in Express settings. Maximum intensity projections were created for quantitative image analysis using the general analysis toolkit of NIS Elements software.

### Widefield Ca^2+^ imaging:

Widefield Ca^2+^ imaging was conducted on muscle 4 NMJs using a Zeiss Axio Examiner A1 upright fixed-stage microscope equipped with a pco.panda 4.2 sCMOS camera (Excelitas) in 1.8 mM Ca^2+^ HL3 saline. High-power 470 nm LED (Thorlabs, M470L4) was used for illumination with a 63x/1.0 NA water-dipping lens, with image acquisition controlled using Nikon NIS-Elements software. Regions of interest (ROIs) were defined to encompass terminal boutons of MN-Ib branches on muscle 4. Images were acquired at 256 × 256 pixels with a frame rate of 100 fps. Time-series imaging was performed for 60 secs without external stimulation to capture spontaneous quantal Ca^2+^ events. For simultaneous electrophysiological recordings, Clampex software (Molecular Devices) was employed to perform current-clamp recordings in gap-free mode. A digital TTL trigger signal from Clampex synchronized the imaging acquisition in NIS-Elements, ensuring precise temporal alignment between electrophysiological and imaging data. Data was collected from a minimum of six biological replicates per genotype.

### STED imaging:

STED super-resolution microscopy was performed as previously described(He et al., 2023, Chien et al., 2025). Briefly, STED imaging was performed with an Abberior STEDYCON system mounted on a Nikon Eclipse FN1 upright microscope. The system is equipped with four excitation lasers (640, 561, 488 and 405 nm), a pulsed STED laser at 775 nm, and three avalanche photodiode detectors operating in a single photon counting mode. Alexa Fluor 488-conjugated anti-HRP was used to locate NMJs, whereas Abberior STAR Red and Alexa Fluor 594 secondary dyes were used for the STED channels. Depletion was performed at 775 nm with time-gated detection set to open from 1 ns to 7 ns after each excitation pulse. Emission was detected with two avalanche photodiodes (band-pass filters: 600 ± 25 nm for STAR RED and 675 ± 25 nm for Alexa Fluor 594). Images were acquired sequentially with a pixel dwell time of 10 μs and 3× line accumulation. Multichannel z-stack STED images were acquired using a 100× Nikon Plan APO 1.45 NA oil immersion objective with a 20-nm fixed pixel size at 130 nm steps, which yields an effective lateral resolution of ~60 nm. Each image covered one to two boutons (25-64 μm^2^) on muscle 4 of segment A3. Raw STED images were corrected for thermal drift and channel crosstalk, then deconvolved in SVI Huygens software Essential 24.04.0 (Scientific Volume Imaging B.V.) using theoretical STED point-spread and default iteration settings of the Good’s MLE algorithm.

### CaFire program:

CaFire is a software tool designed for the analysis and processing of Ca^2+^ imaging data, compatible with both raw intensity data and ΔF/F normalized data. The graphical user interface (GUI) of CaFire is implemented with the Python Tkinter library, while the visualization panel is powered by Matplotlib. The method of automated peak detection utilizes signal processing functions from SciPy (Virtanen et al., 2020). Peaks that are not detected automatically can be selected manually. CaFire identifies the nearest peak to the location where the user clicks. For peak quantification analysis, CaFire calculates rise and decay properties by performing curve fitting on data preceding and following the peak, respectively. The curve fitting is performed using the Levenberg–Marquardt gradient descent algorithm, implemented in the curve fit function of SciPy (Virtanen et al., 2020). The rise time constant is calculated by applying an exponential fit to the rise phase (Shemesh et al., 2020). The decay is modeled using a natural exponential decay function: $y=y_{peak}\cdot e^{-t∕\tau_{decay}}$ (Fleming et al., 2021). The source code and a Windows executable are available for download on CaFire’s GitHub repository (https://github.com/linj7/CaFire) and has been archived with a DOI at Zenodo: 10.5281/zenodo.1552996. The software is released under the MIT License.

### Statistical analysis:

Data were analyzed using NIS Elements software (Nikon), MiniAnalysis (Synaptosoft), SVI Hugyens Essential (version 22.10), CaFire (https://github.com/linj7/CaFire), GraphPad Prism (version 10.0), and Microsoft Excel software (version 16.22). Sample values were tested for normality using the D’Agostino & Pearson omnibus normality test which determined that the assumption of normality of the sample distribution was not violated. Data were then compared using either a one-way ANOVA and tested for significance using a Tukey’s multiple comparison test or using an unpaired two-tailed Student’s t-test with Welch’s correction. In all figures, error bars indicate ±SEM, with the following statistical significance: p<0.05 (*), p<0.01 (**), p<0.001 (***), p<0.0001 (****); ns=not significant. Additional statistical details for all experiments are summarized in Table S1.

## Supplementary Material

1
