# Supplementary material for: Resolving synaptic events using subsynaptically targeted GCaMP8 variants
Source: bioRxiv. 2025 Jun 19:2025.06.19.660577. Preprint. [Version 1] doi: 10.1101/2025.06.19.660577 (PMC12224541; doi:10.1101/2025.06.19.660577)
Supplement: 1 [file NIHPP2025.06.19.660577v1-supplement-1.pdf]

bioRxiv preprint doi: https://doi.org/10.1101/2025.06.19.660577; this version posted June 19, 2025. The copyright holder for this preprint (which was not certified by peer review) is the author/funder, who has granted bioRxiv a license to display the preprint in perpetuity. It is made available under aCC-BY 4.0 International license.

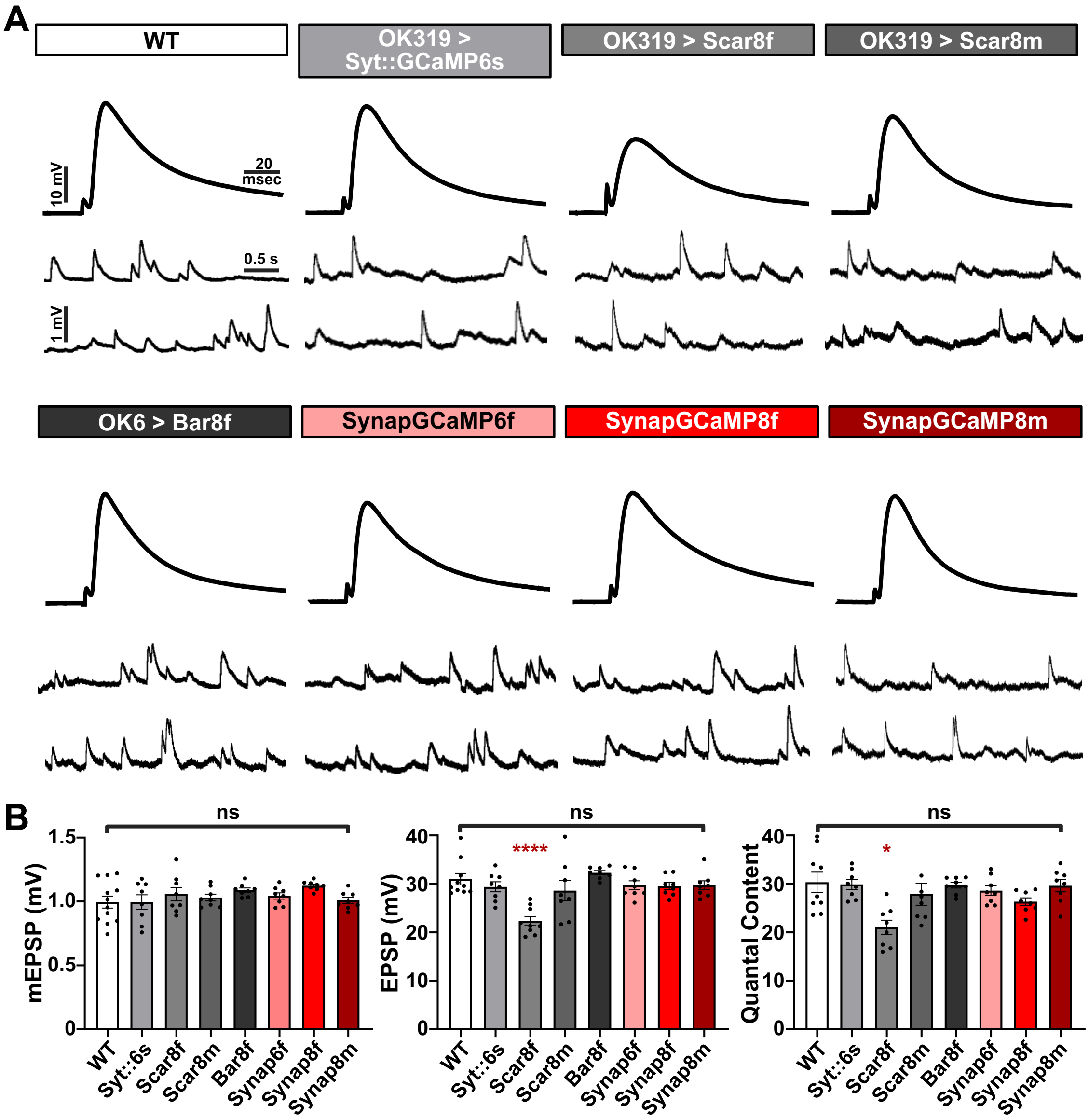

Supplemental Figure S1: GCaMP expression does not perturb synaptic transmission at the Drosophila NMJ. (A) Representative electrophysiological traces in the indicated genotypes showing evoked excitatory postsynaptic potentials (EPSPs, top traces) and spontaneous miniature events (mEPSPs, bottom traces) from muscle 6. **(B)** Quantification of synaptic parameters: average mEPSP amplitude (left), evoked EPSP amplitude (middle), and quantal content (right). No significant differences in mEPSP, EPSP, or quantal content values are observed in genotypes expressing the indicated sensor with the exception of Scar8f, where a significant reduction in EPSP amplitude and quantal content was observed. Data are presented as mean ±SEM with individual data points shown. Error bars represent ±SEM. Detailed statistics including p-values are provided in Table S1.

Supplementary Table 1: Absolute values and statistical comparisons for Ca^{2+} imaging and electrophysiology data.
This table reports the full statistical details and properties for data presented in the indicated figures, including p-values, mean ± SEM, sample sizes (n), and genotypes for all conditions tested. Ca^{2+} imaging parameters include ΔF/F (or ΔR/R), rise time (τ_{rise}), and decay time (τ^{decay}) constants. Electrophysiological parameters include mEPSP amplitude, EPSP amplitude, quantal content (QC), input resistance, and resting potential. p-values from one-way ANOVA with Tukey's multiple comparison test are shown for key contrasts between genotypes and indicators. Data for outlier analysis, correlation analysis and linear regression analyses are included where applicable.

Figure	Label	Genotype	Motor Neuron	△F/F (△R/R)	p value (6s vs 8f, 6s vs 8m, 8f vs 8m)	Tau rise (msec)	p value (6s vs 8f, 6s vs 8m, 8f vs 8m)	Tau decay (msec)	p value (6s vs 8f, 6s vs 8m, 8f vs 8m)	n
3E	Syt::GCaMP6s	w;OK319-GAL4/+; UAS-Syt::GCaMP6s/+	Ib	0.138 (±0.004)	<0.0001 (****), <0.0001 (****), <0.0001 (****)	43.77 (±3.134)	<0.0001 (****), <0.0001 (****), 0.7827 (ns)	171.7 (±8.01)	<0.0001 (****), <0.0001 (****), 0.0046 (**)	14
3E	Scar8f	w;OK319-GAL4/+; UAS-Syt::mScarlet::GCaMP8f/+	Ib	0.389 (±0.010)		5.265 (±1.165)		66.64 (±1.849)		15
3E	Scar8m	w;OK319-GAL4/+; UAS-Syt:: mScarlet3::GCaMP8m/+	Ib	0.632 (±0.011)		6.984 (±0.604)		99.20 (±3.785)		14

Figure	Label	Genotype	Motor Neuron	△F/F (△R/R)	p value	n
4D	Scar8m/WT Ib	*w;OK319-GAL4/+; UAS-Syt:: mScarlet3::GCaMP8m/+*	Ib	0.590 (±0.048)	<0.0001 (****)	9
4D	Scar8m/WT Is	*w;OK319-GAL4/+; UAS-Syt:: mScarlet3::GCaMP8m/+*	Is	1.100 (±0.060)		9
4F	Scar8m/WT Ib	w;OK319-GAL4/+; UAS-Syt:: mScarlet3::GCaMP8m/+	Ib	0.605 (±0.042)	<0.0001 (****)	9
4F	GluRIIA$^{-/-}$ Ib	*W;OK371,GluRIIA^{pv3}/GluRIIA^{pv3}; UAS-Syt:: mScarlet3::GCaMP8m/+*	Ib	0.941 (±0.035)		12

Figure 5D: Data represent mean ± SEM of ΔR/R values for 29 active zones from Bar8f-expressing NMJs (genotype: *w;OK6-GAL4/+; BRP::mScarlet::GCaMP8f/+*) (n=15). Outlier status is determined using the interquartile range (IQR) method on column means: Q1 = 1.6863 (25th percentile), Q3 = 2.0714 (75th percentile), IQR = 0.3851, lower bound = Q1 - 1.5 × IQR = 1.10865, upper bound = Q3 + 1.5 × IQR = 2.64905. Only column 26 (mean = 2.7153) exceeds the upper bound and is marked as an outlier.

**AZ#**	**1**	**2**	**3**	**4**	**5**	**6**	**7**	**8**	**9**	**10**
**△R/R**	2.0653 (±0.1227)	1.7817 (±0.1678)	1.5654 (±0.1008)	1.5108 (±0.0748)	1.5480 (±0.1160)	1.7392 (±0.1039)	1.6863 (±0.0871)	1.8305 (±0.0725)	1.7421 (±0.1205)	1.6387 (±0.0883)
**Outlier**	No	No	No	No	No	No	No	No	No	No
**AZ#**	10	12	13	14	15	16	17	18	19	20
**△R/R**	1.7010 (±0.0911)	1.8863 (±0.1897)	1.6653 (±0.1493)	2.0600 (±0.2155)	2.4527 (±0.2143)	2.0714 (±0.1329)	1.8797 (±0.1354)	2.3911 (±0.1772)	1.7171 (±0.1172)	1.6114 (±0.0969)
**Outlier**	No	No	No	No	No	No	No	No	No	No
**AZ#**	21	22	23	24	25	26	27	28	29	
**△R/R**	1.7301 (±0.0965)	2.3996 (±0.1657)	1.4464 (±0.1430)	1.8058 (±0.1455)	1.9327 (±0.1239)	2.7153 (±0.1308)	2.1330 (±0.1377)	2.1338 (±0.1337)	2.1508 (±0.1789)	
**Outlier**	No	No	No	No	No	**Yes**	No	No	No	

Figure	Label	Genotype	Motor Neuron	Metric (Y vs X)	Equation	Pearson's r	R^2	p-value (correlation)	n
5E	Bar8f	*w;OK6-GAL4/+; BRP::mScarlet::GCaMP8f/+*	Ib	ΔR/R vs. AZ Size	Y = -0.8644*X + 2.355	-0.4902	0.2403	0.0069 (**)	29
5F	Bar8f	*w;OK6-GAL4/+; BRP::mScarlet::GCaMP8f/+*	Ib	Sum ΔF vs. AZ Size	Y = 259.8*X + 40.40	0.7316	0.5353	<0.0001 (****)	29

Figure	Label	Genotype	Motor Neuron	△F/F	p value (6s vs 8f, 6s vs 8m, 8f vs 8m)	Tau rise (msec)	p value (6s vs 8f, 6s vs 8m, 8f vs 8m)	Tau decay (msec)	p value (6s vs 8f, 6s vs 8m, 8f vs 8m)	n
6E	SynapGCaMP6f	*w;MHC-CD8-GCaMP6f-Sh;+*	Ib	0.271 (±0.024)	0.2164 (ns), <0.0001 (****), <0.0001 (****)	21.09 (±2.514)	0.0182 (*), 0.0070 (**), 0.9944 (ns)	98.76 (±6.914)	<0.0001 (****), <0.0001 (****), 0.0036 (**)	47
6E	SynapGCaMP8f	*w;;MHC-CD8-GCaMP8f-Sh*	Ib	0.349 (±0.030)		14.04 (±1.190)		41.98 (±1.829)		50
6E	SynapGCaMP8m	*w;;MHC-CD8-GCaMP8m-Sh*	Ib	0.575 (±0.043)		14.22 (±0.985)		67.35 (±1.640)		58

Figure	Label	Genotype	Motor Neuron	% mEPSPs detected as Ca^{2+} minis	p value (6s vs 8f, 6s vs 8m, 8f vs 8m)	n
7B	SynapGCaMP6f	*w;MHC-CD8-GCaMP6f-Sh;+*	Ib	56.87 (±7.217)	<0.0001 (****), <0.0001 (****), 0.5645 (ns)	12
7B	SynapGCaMP8f	*w;;MHC-CD8-GCaMP8f-Sh*	Ib	87.55 (±1.538)		17
7B	SynapGCaMP8m	*w;;MHC-CD8-GCaMP8m-Sh*	Ib	93.08 (±1.318)		12

Figure	Label	Genotype	Motor Neuron	Metric (Y vs X)	Equation	Pearson's r	R^2	p-value (correlation)	n
7C	SynapGCaMP6f	w;MHC-CD8-GCaMP6f-Sh;+	Ib	Ca^{2+} mini ΔF/F vs. mEPSP (mV)	Y = 0.01231*X + 0.008230	0.4579	0.2097	<0.0001 (****)	83
7C	SynapGCaMP8f	w;;MHC-CD8-GCaMP8f-Sh	Ib	Ca^{2+} mini ΔF/F vs. mEPSP (mV)	Y = 0.02910*X + 0.01663	0.7316	0.5374	<0.0001 (****)	98
7D	SynapGCaMP8m	w;;MHC-CD8-GCaMP8m-Sh	Ib	Ca^{2+} mini ΔF/F vs. mEPSP (mV)	Y = 0.04216*X + 0.01672	0.8098	0.6557	<0.0001 (****)	97

Figure	Label	Genotype	Motor Neuron	mEPSP amplitude (mV)	mEPSP frequency (Hz)	R input (MΩ)	Resting potential (mV)	P Value (significance: mEPSP, mEPSP freq)	n
7E	WT	w;;Is-GAL4/UAS-BoNT-C	Ib	0.636 (±0.015)	1.062 (±0.100)	11.91 (±0.115)	64.45 (±1.561)	-	41
7E	IIB$^{-/-}$	w;GluRIIB^{sp5};Is-GAL4/UAS-BoNT-C	Ib	0.824 (±0.017)	2.433 (±0.147)	12.89 (±0.197)	65.46 (±1.541)	<0.0001 (****), <0.0001 (****)	41
7E	IIA$^{-/-}$	w;GluRIIA^{pv3};Is-GAL4/UAS-BoNT-C	Ib	0.383 (±0.007)	0.715 (±0.111)	12.11 (±0.138)	65.38 (±1.19)	<0.0001 (****), <0.0001 (****)	41

Figure	Label	Genotype	Motor Neuron	△F/F	p value (WT vs IIB-/-, WT vs IIA-/-, IIB-/- vs IIA-/-)	n
7E	WT	*w;;MHC-CD8-GCaMP8m-Sh*	Ib	0.713 (±0.021)	<0.0001 (****), <0.0001 (****), <0.0001 (****)	65
7E	IIB$^{-/-}$	*W;GluRIIBsp5;MHC-CD8-GCaMP8m-Sh*	Ib	0.967 (±0.036)		69
7E	IIA$^{-/-}$	*W;GluRIIBsp5;MHC-CD8-GCaMP8m-Sh*	Ib	0.427 (±0.017)		59

Kolmogorov–Smirnov Test Results for Mini Amplitude Distributions for MN-Ib

D statistic: Maximum difference between cumulative distributions.

All comparisons reached $p < 0.001$, indicating significantly different mini amplitude distributions.

Figure	Label	Genotype	Motor Neuron	mEPSP amplitude (mV)	D statistic (WT vs IIB-/-, WT vs IIA-/-, IIB-/- vs IIA-/-)	P Value (WT vs IIB-/-, WT vs IIA-/-, IIB-/- vs IIA-/-)	n
7F	WT	w;;Is-GAL4/UAS-BoNT-C	Ib	0.636 (±0.015)	0.1854, 0.3647, 0.4043	<0.0001 (****), <0.0001 (****), <0.0001 (****)	41
7F	IIB$^{-/-}$	w;GluRIIB^{sp5};Is-GAL4/UAS-BoNT-C	Ib	0.824 (±0.017)			41
7F	IIA$^{-/-}$	w;GluRIIA^{pv3};Is-GAL4/UAS-BoNT-C	Ib	0.383 (±0.007)			41

Figure	Label	Genotype	Motor Neuron	△F/F	D statistic (WT vs IIB-/-, WT vs IIA-/-, IIB-/- vs IIA-/-)	p value (WT vs IIB-/-, WT vs IIA-/-, IIB-/- vs IIA-/-)	n
7F	WT	*w;;MHC-CD8-GCaMP8m-Sh*	Ib	0.713 (±0.021)	0.2046, 0.2938, 0.3277	<0.0001 (****), <0.0001 (****), <0.0001 (****)	65
7F	$IIB^{-/-}$	*W;GluRIIB^{sp5};MHC-CD8-GCaMP8m-Sh*	Ib	0.967 (±0.036)			69
7F	$IIA^{-/-}$	*W;GluRIIB^{sp5};MHC-CD8-GCaMP8m-Sh*	Ib	0.427 (±0.017)			59

Figure	Label	Genotype	Motor Neuron	mEPSP amplitude (mV)	EPSP amplitude (mV)	QC	mEPSP frequency (Hz)	R input (MΩ)	Resting potential (mV)	n	P value (significance: mEPSP, EPSP, QC)
S1B	WT	*w^{1118}*	Is + Ib	0.995 (±0.048)	30.96 (±1.182)	30.34 (±2.101)	3.133 (±0.319)	12.11 (±0.138)	65.38 (±1.19)	10	-
S1B	OK319> Syt::GCaMP6s	*w;OK319-GAL4/+; UAS-Syt::GCaMP6s/+*	Is + Ib	0.996 (±0.057)	29.39 (±1.035)	29.88 (±1.024)	4.067 (±0.509)	12.89 (±0.197)	65.46 (±1.541)	8	>0.9999 (ns), 0.8985 (ns), 0.9997(ns)
S1B	OK319> Scar8f	*w;OK319-GAL4/+; UAS-Syt::mScarlet::GCaMP8f/+*	Is + Ib	1.057 (±0.053)	22.33 (±0.941)	21.02 (±1.488)	2.222 (±0.4923)	12.71 (±0.126)	66.62 (±1.284)	9	0.7858 (ns), <0.0001 (****), <0.0001 (***)
S1B	OK319> Scar8m	*w;OK319-GAL4/+; UAS-Syt::mScarlet3::GCaMP8m/+*	Is + Ib	1.031 (±0.027)	28.61 (±2.122)	27.89 (±2.289)	2.692 (±0.187)	10.87 (±0.108)	66.23 (±1.105)	8	0.9867 (ns), 0.5656 (ns), 0.7704 (ns)
S1B	OK319> Bar8f	*w;OK319-GAL4/+; BRP::mScarlet::GCaMP8f/+*	Is + Ib	1.088 (±0.018)	32.30 (±0.425)	29.73 (±0.595)	2.117 (±0.611)	11.25 (±0.143)	63.62 (±1.443)	8	0.3526 (ns), 0.9535 (ns), >0.9999 (ns)
S1B	SynapGCaMP6f	*w;MHC-CD8-GCaMP6f-Sh;+*	Is + Ib	1.043 (±0.025)	29.69 (±0.934)	28.60 (±1.011)	3.606 (±0.608)	11.84 (±0.198)	65.13 (±1.484)	8	0.9317 (ns), 0.9646 (ns), 0.9495 (ns)
S1B	SynapGCaMP8f	*w;;MHC-CD8-GCaMP8f-Sh*	Is + Ib	1.124 (±0.013)	29.55 (±0.736)	26.34 (±0.781)	2.692 (±0.390)	13.97 (±0.160)	66.51 (±1.471)	8	0.0825 (ns), 0.9395 (ns), 0.2607 (ns)
S1B	SynapGCaMP8m	*w;;MHC-CD8-GCaMP8m-Sh*	Is + Ib	1.007 (±0.024)	29.73 (±0.901)	28.60 (±1.011)	3.587 (±0.546)	11.90 (±0.105)	67.39 (±1.276)	8	>0.9999 (ns), 0.9702 (ns), 0.9998 (ns)

KEY RESOURCES TABLE

bioRxiv preprint doi: https://doi.org/10.1101/2025.06.19.660577; this version posted June 19, 2025. The copyright holder for this preprint (which was not certified by peer review) is the author/funder, who has granted bioRxiv a license to display the preprint in perpetuity. It is made available under aCC-BY 4.0 International license.

REAGENT/RESOURCE		SOURCE	IDENTIFIER
**Antibodies**	**Dilution**		
Mouse anti-DLG	1:50	Developmental Studies Hybridoma Bank (DSHB)	4F3
Mouse anti-BRP	1:100	DSHB	nc82
Chicken anti-GFP	1:1000	Aves Lab	GFP-1010
Rabbit anti-GluRIIC	1:100	(Goel & Dickman, 2018)	
Rabbit anti-SYT	1:50	(Mackler et al., 2002)	
Alexa Fluor 488 conjugated Donkey anti-chicken secondary antibody	1:400	Jackson ImmunoResearch	703-545-155
Cy3-conjugated Donkey anti-rabbit secondary antibody	1:400	Jackson ImmunoResearch	711-165-152
Alexa Fluor 647 conjugated Donkey anti-mouse secondary antibody	1:400	Jackson ImmunoResearch	715-605-150
DyLight 405 conjugated Donkey anti-mouse secondary antibody	1:400	Jackson ImmunoResearch	715-475-150
Alexa Fluor 647 conjugated Donkey anti-rabbit secondary antibody	1:400	Jackson ImmunoResearch	711-605-152
**Drosophila Strains**			
UAS-SYT::mScarlet::GCaMP8f (Scar8f)		(Li et al., 2021)	
UAS-SYT::mScarlet3::GCaMP8m (Scar8m)		(This paper)	
UAS-SYT::GCaMP6s		BDSC	64415
SynapGCaMP6f		(Newman et al., 2017)	
SynapGCaMP8f		(Han et al., 2022)	
SynapGCaMP8m		(This paper)	
UAS-BRP::mCherry::GCaMP6s		(Kiragasi et al., 2017)	
UAS-BRP::mScarlet::GCaMP8f (Bar8f)		(This paper)	
OK6-GAL4		(Aberle et al., 2002)	
OK319-GAL4		(Sweeney et al., 1995)	
UAS-BoNT-C		(Han et al., 2022)	
R27E09-GAL4 (Is-Gal4)		BDSC	49227
w^{1118}		BDSC	5905
GluRIIA^{pv3}		(Han et al., 2023)	
GluRIIB^{sp5}		(Han et al., 2023)	
**Software and Algorithms**			
ImageJ		https://imagej.net/	1.8.0
NIS-Elements software		Nikon Instruments	5.41.02 (Build 1711)
Mini Analysis		Synaptosoft	6.0.7

bioRxiv preprint doi: https://doi.org/10.1101/2025.06.19.660577; this version posted June 19, 2025. The copyright holder for this preprint (which was not certified by peer review) is the author/funder, who has granted bioRxiv a license to display the preprint in perpetuity. It is made available under aCC-BY 4.0 International license.

Axon pCLAMP Clampfit		Molecular Devices	10.7
Clampex		Molecular Devices	10.7
GraphPad Prism		GraphPad	10.0.1
Jupyter Notebook		Anaconda	6.0.1
Python		https://www.python.org/	3.10.11
Excel		Microsoft	2021
CaFire		(This paper)	

REFERENCES

Aberle, H., Haghighi, A. P., Fetter, R. D., McCabe, B. D., Magalhães, T. R., & Goodman, C. S. (2002). wishful thinking encodes a BMP type II receptor that regulates synaptic growth in Drosophila. *Neuron*, *33*(4), 545-558. https://doi.org/10.1016/s0896-6273(02)00589-5

Goel, P., & Dickman, D. (2018). Distinct homeostatic modulations stabilize reduced postsynaptic receptivity in response to presynaptic DLK signaling. *Nature communications*, *9*(1), 1856-1814. https://doi.org/10.1038/s41467-018-04270-0

Han, Y., Chien, C., Goel, P., He, K., Pinales, C., Buser, C., & Dickman, D. (2022). Botulinum neurotoxin accurately separates tonic vs. phasic transmission and reveals heterosynaptic plasticity rules in Drosophila. *eLife*, *11*. https://doi.org/10.7554/eLife.77924

Han, Y., Goel, P., Chen, J., Perry, S., Tran, N., Nishimura, S., Sanjani, M., Chien, C., & Dickman, D. (2023). Excess glutamate release triggers subunit-specific homeostatic receptor scaling. *Cell Reports*.

Kiragasi, B., Wondolowski, J., Li, Y., & Dickman, D. K. (2017). A Presynaptic Glutamate Receptor Subunit Confers Robustness to Neurotransmission and Homeostatic Potentiation. *Cell Rep*, *19*(13), 2694-2706. https://doi.org/10.1016/j.celrep.2017.06.003

Li, X., Chien, C., Han, Y., Sun, Z., Chen, X., & Dickman, D. (2021). Autocrine inhibition by a glutamate-gated chloride channel mediates presynaptic homeostatic depression. *Science advances*, *7*(49), eabj1215-eabj1215. https://doi.org/10.1126/sciadv.abj1215

Mackler, J. M., Drummond, J. A., Loewen, C. A., Robinson, I. M., & Reist, N. E. (2002). The C(2)B Ca(2+)-binding motif of synaptotagmin is required for synaptic transmission in vivo. *Nature*, *418*(6895), 340-344. https://doi.org/10.1038/nature00846

Newman, Z. L., Hoagland, A., Aghi, K., Worden, K., Levy, S. L., Son, J. H., Lee, L. P., & Isacoff, E. Y. (2017). Input-Specific Plasticity and Homeostasis at the Drosophila Larval Neuromuscular Junction. *Neuron*, *93*(6), 1388-1404.e1310. https://doi.org/10.1016/j.neuron.2017.02.028

Sweeney, S. T., Broadie, K., Keane, J., Niemann, H., & O'Kane, C. J. (1995). Targeted expression of tetanus toxin light chain in Drosophila specifically eliminates synaptic transmission and causes behavioral defects. *Neuron*, *14*(2), 341-351. https://doi.org/10.1016/0896-6273(95)90290-2
